# Illinois State Dental Association, 1873

**Published:** 1873-10

**Authors:** S. P. Cutler

**Affiliations:** Memphis, Tenn.


### ILLINOIS STATE DENTAL ASSOCIATION, 1873.

BY S. P. CUTLER, M. D., D. D. S., MEMPHIS, TENN.

This very interesting and instructive meeting presented and discussed many subjects of deep interest to the profession.

On page 61 of the proceedings, the writer speaking of the oral secretions and their action on the teeth, says: "The neutrality, or acidity, might be brought about by the carbonic acid which passes off from the system, in large quantities, by each exhalation of air from the lungs. This coming in contact with every portion of the saliva as soon as it is emitted into the mouth, would tend to transform it immediately to an acid fluid."

It is true a certain amount of this acid enters into solution in the oral secretions, mechanically or by a law of gaseous condensation, in water and other liquids. It is this acid that gives to well and spring water that delicious and palatable taste which is wanting in distilled water, or water slightly warmed, as when given for emetic purposes. When taken in large quantities it acts most promptly, more especially if assisted by poking the finger down the throat, when we wish to accomplish this most delectable and desirable end. If we have a snake or any other disgusting thing in our *bread basket*, we have in this a first class emetic, without the aid of the *puke man*, (water, saturated with the acid gas, is not acid water, but neutral,) whether it be our's or that of some one else; unlike our friend on the street car we take no offense at the suggestion of running the finger down the throat for relief; but to return to our subject:

Water absorbs a certain per cent of carbonic acid, and no more, varying at different temperatures, cold water absorbs its own bulk about, the saliva may condense a little more than pure water at the same temperature. Now, this amount of carbonic acid gas condensed and dissolved in the oral secretions, can produce no chemical change, any more than in the case of water. As a little heat rapidly expels the acid gas, even at the temperature of the body, the capacity of water for this gas is almost *nil*, and the same rule ought to hold good in the case of the oral secretions. The gas in a liquid form, when dissolved or diluted in water, can be driven off by heat alone, but must be neutralized by an alkali, or some other base, before removal, or by evaporation of the water; in some instances, as in oxalic acid, and some others, leaving the acid behind, their affinities for water being weak.

Same page, lower down, he says: "The saliva now being chemically changed, the conditions favorable to the solution of the phosphate of lime of course are not present, and this substance is precipitated as an insoluble powder. It now finds its way between the margin of the gums, and necks of the teeth," etc. I am unable to determine, how the presence of carbonic acid in the saliva could render the soluble phosphate of lime insoluble, as the carbonic acid certainly cannot decompose the phosphate; if so what would be the result of the constant presence in the blood and tissues of carbonic acid in large quantities, coming in contact everywhere with salts of lime, to supply the bones and teeth through the circulation? Certainly disastrous. Admitting, as the author suggests, the possibility of the presence of carbonic acid, producing an insoluble powder of the phosphate, what would be the consequence? We would expect to find calculi deposited throughout the entire organism, wherever a lodgment could take place. This subject needs more extensive investigation, as we are yet in the twilights only, on this subject. This paper was not discussed to the extent it might have been, with additional profit to the profession.

Page 63, the speaker said: "In reference to absorption of the walls of the pulp chamber, that it is owing to inflammation, swelling of the pulp and consequent pressure, but he could not see where any such influences could act on the outer portion of the enamel," (speaking of erosions.)

I was not aware before the above statements, that a pulp chamber ever became enlarged while a live pulp existed therein, neither can I imagine how any one can determine this fact, as there are no chances to make definite comparisons of size, before and after the death of the pulp. Neither can I imagine how the pressure of an inflamed pulp, could produce absorption of pulp chamber. How far can these statements be demonstrated or proven?

On same page, another speaker in referring to *vital action*, carrying away dentine under fillings, exposing pulps, does not award any agency to the action the filling may have on the absorption of dentine, by electric, galvanic, or other influence as physical agencies, solving the lime salts by removing polarity of the inorganic atoms, or in some other manner, not yet fully understood.

On pages 82 to 85, on the subject of utilization of the lime salts of the absorbed fangs of deciduous teeth to the formation the permanent ones, I have not quoted verbatim. The writer seems to be satisfied that he has given us sufficient evidence by reasoning and quotations from standard authors to establish such facts. I cannot find, by his reasoning and arguments, how he reutilizes these materials, only the broad assertion of the facts. I only inquire, for information, how these changes are brought about, as a matter important to physiological enquiry. We all want to know how these changes take place, and then the proofs, then we are enlightened and edified. Somehow or other, I had got the idea into my head, otherwise. I believe the writer himself has stated that the lime salts do not pass off by the urine ; of course, that must be a surplus, more than was needed in the economy. So far as the argument concerning common salt is concerned, I cannot see the facts in the same light as he does, as salt never, I believe, becomes a component of any solid tissue like teeth and bones, but enters into combinations of a less stable nature, more especially the effect of chemical, rather than acid affinities. And from the evidences adduced, I am unable to see where he has reproduced common salt from the same elements that had been decomposed as common salt. As to muscular and other organic quaternary compounds being again suited to nutrition, we all agree ; more especially, in our beefsteak we take for dinner, and all other dead meats, as we cannot eat live meats. In the first place, our beefsteak has never been decomposed and converted into any other group of organic compounds, and is in a proper condition to become pabulum for nutrition, like all animal and vegetable food, and must undergo a process of digestion or assimilation to a certain extent.

Let me ask if a muscle in our bodies, ever by natural processes becomes broken up in any way, other than by starvation, disease, or exercise in health ? then only by oxydation, and forming again binary compounds, as carbonic acid, water and ammonia, out of the chief organic elements. The same may be asked of all the elements of organic life.

On page 90, speaking of cementum, the speaker says, "There is quite an intimate relationship existing between dentine and true bone. The most striking analogy is shown by the existence of Haversian canals in both, and the matter entering into their composition is quite similar." This is certainly something new to me: the existence of Haversian canals in true dentine. This is a sight I have never seen under the microscope. This statement may have been an oversight of the speaker, or a misprint.

On page 92, same speaker says, "It is claimed by some that amalgam may be driven with such force into a cavity as to cause the minute globules of mercury to insinuate themselves into the freshly exposed tubuli, thus creating inflammation of the dentine, and ultimately destroy the vitality of the tooth. I am not prepared yet to believe in inflammation of dentine, as there are no true blood-vessels, or red blood in the tubuli of the dentine.

On page 91, same speaker says, "A sandarac plug not only makes a temporarily water-proof filling, but it forms a coating over the walls of the cavity, thereby closing up to some extent the ends of the tubuli, when, at a future time, these tubes are effectually closed by deposits of lime salts."

Now right here, I certainly must take issue with the ideas advanced, as I cannot see how any such closing up of the tubuli can take place by lime salts, after removal of pulp, and the elements of internal tooth formation and change gone, until Gabriel shall blow his last trump, and the dead bones come to life again; and then by old age, wear, decay or other weakening causes, the tubuli may become more or less obliterated, while dentinal fibrils there exist, and finally become obliterated. as the filling up process goes on *pari passu*; and in no other way, so far as we know with all the lights before us.

On page 169, in speaking of American dentists, says, "It is a gratifying fact, that American dentists, as operators, stand in the front rank, yet we are indebted to European observers for *all* our *scientific* facts." This statement, I think, is unfounded, so far as the latter clause is concerned. I think the European dentists are quite as much indebted to us as we are to them for the light and knowledge of every kind pertaining to the profession. We have more standard text-books than all Europe put together, and quite as scientific and more dental literature (aside from the standard books) in our journals. In all other respects I agree heartily with him.

